# Preliminary Assessment of Leisure Horses’ Preferences for Different Forms of Carrot (*Daucus carota* subsp. *sativus*)

**DOI:** 10.3390/ani15233385

**Published:** 2025-11-24

**Authors:** Anna Mańkowska, Barbara Maria Dobraczyńska, Joanna Szewczak, Zofia Chodup, Bartosz Radzanowski, Ivan Matychyn, Dorota Witkowska

**Affiliations:** 1 Department of Animal Welfare and Research, Faculty of Animal Bioengineering, University of Warmia and Mazury in Olsztyn, 10-719 Olsztyn, Poland; barbara.dobraczynska@student.uwm.edu.pl (B.M.D.); joanna.szewczak@student.uwm.edu.pl (J.S.); zofia.chodup@student.uwm.edu.pl (Z.C.); dorota.witkowska@uwm.edu.pl (D.W.); 2 Faculty of Mathematics and Computer Science, University of Warmia and Mazury in Olsztyn, 10-710 Olsztyn, Poland; bartosz.radzanowski@uwm.edu.pl (B.R.); matychyn@matman.uwm.edu.pl (I.M.)

**Keywords:** carrots, feed texture, dietary preferences, equine welfare

## Abstract

Cultivated carrot (*Daucus carota* subsp. *sativus*) is a valuable component of equine diets due to its high β-carotene content (a precursor of vitamin A) and desirable sensory properties. However, its use may be limited by short shelf life, susceptibility to spoilage, and firm texture, which can be challenging for horses with dental problems. This study aimed to evaluate horses’ preferences for different physical forms of carrots. Twenty-one leisure horses aged 3–22 years were individually evaluated in a free-choice test. Each horse was simultaneously offered equal portions (150 g) of five carrot forms: raw, grated, boiled, dried, and juice. Feeding time and selection order were recorded, and data were analyzed using non-parametric tests. The form of carrots had a significant effect on feeding time (*p* < 0.001). Mean feeding time differed among forms; boiled carrots were consumed in 78.4 ± 15.3 s, whereas dried carrots required 156.2 ± 28.7 s. Among solid forms, raw carrots were most frequently selected first, followed by grated and dried carrots, while boiled carrots were the least preferred (*p* < 0.01). Carrot juice was initially rejected by most horses, but its acceptability increased in later trials. No significant correlations were found between horse age and either feeding time or selection order. The findings emphasize the practical importance of carrot processing in improving feed management and meeting horses’ individual nutritional needs.

## Simple Summary

Carrots are a popular supplement to equine diets, serving as a source of beta-carotene and other valuable nutrients. However, the main disadvantage of carrots is that they spoil easily and cannot be stored for long periods of time. In addition, carrots require chewing, and their hardness can present difficulties for older horses with dental issues. Therefore, the administration of processed rather than raw carrots may be a more practical option in some circumstances. The aim of this study was to determine the physical form of carrots that is most acceptable to horses. Carrots were offered in 5 forms—raw, grated, boiled, dried, and as juice—to 21 leisure horses. Carrot intake by horses was clearly affected by the form of presentation. Raw carrots were the most attractive and usually chosen first, while dried carrots took more time to eat. Most horses initially refused carrot juice but showed more interest after repeated exposure, indicating that they can become accustomed to this unfamiliar liquid form. These findings show that carrot processing influences both the eating time and preference of horses. The results highlight that choosing an appropriate form of carrots can help adapt feeding practices to individual horse needs.

## 1. Introduction

Horses require small and frequent portions of feed because the equine digestive system has evolved to process high-fiber forage during continuous grazing [1]. Adequate nutrition that meets the anatomical and physiological needs of the species has significant implications for horse health and performance, as well as maintenance costs [2].

### 1.1. Carrot (Daucus carota *subsp.* sativus) in Horse Nutrition

Cultivated carrot (*Daucus carota* subsp. *sativus*) is a commonly used feed component in equine diets, often classified as roughage due to its high fiber content. It serves as a rich source of β-carotene, vitamins, and minerals, although it is also susceptible to spoilage [3,4,5]. Carrots require considerable mastication, which may be problematic for horses with dental dysfunctions [6]. Before serving, carrots should be cleaned, and the degree of chopping should be adjusted to the individual feeding behavior of each horse to minimize the risk of choke. Horses that chew feed calmly may safely consume larger carrot pieces, whereas in animals that eat hastily or have dental issues, finer processing may be advisable [7,8]. The recommended daily carrot intake for horses should not exceed approximately 2–3 kg, provided that the amount of concentrate feed is simultaneously reduced to maintain an appropriate balance of digestible carbohydrates [9]. Fresh carrots contain around 88–90% water and 3.5–6% simple sugars (sucrose, glucose, and fructose) [10,11], which means that a 3 kg portion provides approximately 105–180 g of sugars. For a 500 kg horse, whose safe upper limit for soluble carbohydrate intake is estimated at less than 2 g/kg per day [2], this quantity remains within a physiologically acceptable range. Therefore, moderate carrot feeding does not pose a metabolic risk if the diet is properly balanced. However, the excessive administration of more than 5–6 kg of carrots per day may supply over 300 g of simple sugars, which could predispose susceptible horses—particularly those with insulin resistance or laminitis—to metabolic disorders. Although carrots have a high-water content, there is no evidence that rapid ingestion of fresh carrots causes diarrhea in healthy horses. Occasional loose feces observed after excessive intake of root crops are usually attributed to the osmotic effect of soluble sugars rather than water content itself [12]. For this reason, gradual diet adaptation and portion control are advisable when introducing fresh carrots into the ration.

#### 1.1.1. Carrot as a Source of Vitamin A

According to data from the United States Department of Agriculture FoodData Central database (FDC ID 170393, 170500, 170491) [13,14,15], the β-carotene content of carrots varies depending on the processing method. Raw carrots contain approximately 835 µg of β-carotene per 100 g fresh matter (FM) expressed as retinol activity equivalents (RAE), which corresponds to 8.35 mg/kg FM or approximately 83 mg/kg dry matter (DM), assuming 10% DM. Dried carrots, with about 90% DM contain up to 3420 µg per 100 g FM equivalent to 34.2 mg/kg FM or about 342 mg/kg DM, while carrot juice, containing roughly 10% DM, provides around 956 µg per 100 g FM, corresponding to 9.56 mg/kg FM or 96 mg/kg DM.

For comparison, a 500 kg horse in moderate work requires approximately 22,500 IU of vitamin A per day, equivalent to 13–15 mg of β-carotene [2]. To meet a dietary requirement of β-carotene, a horse weighing 500 kg would need to consume approximately 1.56–1.80 kg of fresh carrots, although bioavailability is affected by processing and storage. Horses grazing fresh pasture usually obtain sufficient β-carotene from green forage, whereas stabled horses fed primarily hay may require additional sources of provitamin A.

It should be noted that the nutritional value of raw (unprocessed) carrots, including the content of provitamin A and other nutrients, is not affected by grating, but high temperatures can reduce the β-carotene content in carrots, particularly during prolonged boiling in water, with losses of up to 43.6% [16].

#### 1.1.2. Limitations Associated with Carrot Storage

Carrots are widely used in human and animal nutrition; however, their high-water content (≈88–90%) and sensitivity to temperature, light, and humidity contribute to rapid microbial growth and nutrient degradation, including changes in carotenoid composition during storage [17]. During storage, carrots lose firmness, color, taste, and aroma as a result of dehydration, the decomposition of volatile compounds, and oxidation processes. Mechanical damage additionally increases the risk of spoilage, which is why carrots should be stored at a temperature of 0–2 °C and 95–98% humidity. However, their shelf life remains limited even under these conditions [18]. In horse husbandry, carrots are often stored in clamps that stabilize temperature, maintain moisture, and prevent wilting. Carrots can be stored for up to six months in clamps [5]. Carrots are layered with sand to reduce exposure to oxygen and light, slow down microbial growth and degradation of nutrients [19]. Drying is one of the most effective food preservation methods that decreases water activity, limits physicochemical reactions, and conserves bioactive carotenoids, but the condition of carrots at the onset of drying significantly affects the quality of the end product [20]. Carrot juice also has a short shelf life and should be chilled or preserved prior to storage. Thermal processing improves microbial safety, but it may also lead to the loss of vitamin C and carotenoids, and impair the sensory attributes of carrot juice [21].

### 1.2. Challenges in Feeding Carrots to Horses

In horses, dental problems exacerbate with age, and older animals may be affected by tooth loss or the formation of sharp edges on the molars, which can hinder their ability to chew standard feed [12,22]. Dental dysfunctions undermine effective feed grinding and increase the risk of colic and digestive disorders. In addition, digestive efficiency declines with age, and senior horses may require more energy-dense feeds, even at moderate levels of physical activity [12,23]. The feed ration should be balanced and supplemented with vitamins, including vitamin A, and minerals.

Sharp edges or uneven tooth wear can impair food intake in horses. Affected animals may initially show interest in feed, but quickly lose enthusiasm, eat slowly, or refuse to eat. Dental disorders may also result in quidding (dropping partially chewed feed), which is a sign of dysmastication [24]. These symptoms are usually observed in young horses during periods of rapid dental development (between the ages of two and five years) and in older horses whose teeth show age-related wear [25]. To accommodate the needs of horses with dental problems, easy-to-chew and highly digestible products should be incorporated into their diets [26].

Choke is a serious disorder that leads to partial or complete esophageal obstruction by feed particles, such as hay, grain, vegetables, and fruit [27]. Signs of choke include coughing, Signs of choke include coughing, ptyalism, dysphagia, and neck extension colic [28]. Choke is commonly caused by rapid feed intake and dental disorders that impair effective oral processing. These risks can be minimized by avoiding hard treats or large pieces of vegetables and by modifying feed texture, particularly in older horses and animals with dental dysfunctions [29]. In these cases, raw carrots should also be avoided and replaced with processed forms. However, horses respond differently to carrots offered in various forms, which suggests that the texture of carrots affects both palatability and intake.

Recent behavioral studies have provided valuable insights into equine feed palatability and the factors influencing feeding choices. Van den Berg et al. [30] found that odor, taste, and nutrient composition jointly affect feeding behavior, while a related study by the same group [31] showed that horses’ acceptance of novel foods depends strongly on both sensory properties and nutritional balance. DeChant et al. [32] conducted a pilot study evaluating the palatability of a novel feed ingredient using a two-choice preference test in horses. The authors combined quantitative measures such as intake and latency to eat with behavioral observations, including sniffing, head movements, and time spent eating. Horses readily accepted the novel feed, showing minimal avoidance behavior, demonstrating that this simple behavioral design is sensitive enough to detect differences in feed acceptability even in small equine groups. Vinassa et al. [33] conducted a controlled palatability experiment to evaluate how lateralization and temperament affect feed preference in horses. Each animal was simultaneously presented with two feed bowls containing feeds varying in sensory characteristics such as flavor and aroma. Both bowls were placed at the same distance and offered at the same time to eliminate positional bias. Researchers recorded the order in which the horses approached and selected each bowl, the latency to begin eating, and the total time spent at each feed type. The results showed that horses exhibited clear individual preferences, significantly influenced by their behavioral traits and side preferences. Francis et al. [34] compared the palatability of various commercial horse treats using a two-choice design. Horses were offered two treats in separate feed containers, and researchers recorded the order of selection, latency to begin eating, and duration of consumption, in addition to behavioral signs such as sniffing, licking, or turning the head away. This study demonstrated that objective behavioral measures—such as first choice, total intake, and time spent eating—provide a reliable indicator of equine palatability. Springer et al. [35] evaluated the palatability of hempseed meal pellets compared to conventional feed using a standardized two-choice test. Feed acceptance was assessed through order of selection, latency to eat, and time spent at the feed, complemented by qualitative indicators such as approach patterns and willingness to continue feeding. Stachurska et al. [36] investigated horses’ responses to oats supplemented with dried herbs in dry, wet, and wet-sweetened forms. The experiment used a repeated two-choice design, recording feed intake, order of selection, and time spent eating, highlighting how texture and added flavors influence acceptance. Collectively, these studies underscore the importance of sensory and physical properties in determining feed preference.

Horses are naturally grazing animals that spend a substantial portion of their day foraging and consuming feed, typically around 12–13 h under natural conditions, which is essential for maintaining both digestive and mental health [37]. Limiting feeding time or offering feeds that are rapidly ingested can disrupt digestive physiology and natural behavioral patterns, potentially leading to gastrointestinal disturbances or stereotypic behaviors. Root vegetables such as carrots are often used as treats or rewards in horse management, but their physical form can strongly influence feeding behavior, including time spent eating and oral manipulation [37]. Consequently, understanding how different carrot forms are selected and consumed is essential for designing feeding practices that support both nutritional intake and welfare. It provides a foundational framework emphasizing that horses’ feeding behavior is driven not only by nutrient requirements but also by the time spent foraging and the physical act of feed manipulation, which has direct implications for welfare. This evidence underpins the rationale for our study, which focuses on how different carrot forms influence selection order and time spent feeding, thereby linking feed palatability with natural feeding behaviors and welfare considerations. Therefore, the aim of this study was to assess leisure horses’ preferences for different physical forms of carrots.

## 2. Materials and Methods

### 2.1. Animals

The experiment was performed on 21 leisure horses, including 18 mares and three geldings, housed in the Elf Stable in Derc, Poland (53.946314° N, 20.648529° E). The study population included eleven ponies of unknown breed, four Malopolski horses, three Hucul horses, two Friesian horses and one Wielkopolski horse. The horses ranged in age from 3 to 22 years ($\overset{¯}{x}$ = 10.75) and were classified as adult (3–15 years; *N* = 14) or aged (over 16 years; *N* = 7), with a body weight of 300 to 550 kg ($\overset{¯}{x}$ = 370 kg). The animals had ad libitum access to hay in the stalls, green fodder in the paddocks, and water, whereas concentrate feed (oats, soaked beet pulp, wheat bran, beets, and carrots) was served twice daily in the amount adjusted to the individual nutritional requirements of each animal based on the type and level of physical activity, and physiological condition. In their regular concentrate feed, horses received raw, unprocessed carrots, which were neither chopped nor dried. The quantity of carrots was individually adjusted for each horse by the owner and was not modified during this study. The carrots used in the experimental trials were offered separately from the daily feed ration, and all horses had prior exposure only to the raw form of carrots. Additionally, the horses were kept on pasture for approximately 8 h per day. All horses at the stable undergo routine dental examinations performed by a veterinarian every six months, ensuring that no major abnormalities affecting feed intake are present. According to the decision of the Local Ethics Committee for Animal Experimentation in Olsztyn (LKE/07/2025), this study did not require ethical approval.

### 2.2. Research Methods

#### 2.2.1. Preparation of Different Forms of Carrots

The following physical forms of carrots were used in the experiment: raw (unprocessed), grated, boiled, dried, and carrot juice.

Raw carrots were purchased from a farm different from the horses’ regular supplier and were served in unprocessed form (without cutting or thermal processing) to preserve their original consistency and structure. Carrots were thoroughly washed to remove contaminants, soil, and other impurities. To preserve their natural properties, carrots were washed without the use of detergents or other chemical agents.

Grated and boiled carrots, as well as carrot juice, were prepared from portions of raw carrots on the morning of each experimental session and stored in a refrigerator at 6 °C until transport to the stable. This involved approximately 6 h of refrigeration, followed by 1 h of transport at ambient temperature (approximately 20 °C).

Carrots were grated in a Bosch MUM58231 1000 W food processor (Robert Bosch Hausgeräte GmbH, Munich, Germany) equipped with a 5 mm shredding disc, operated at its maximum speed (setting 7) to ensure uniform processing.

Boiled carrots were prepared from a portion of raw carrots, under domestic conditions. Carrots were boiled in water until soft. Cooking time was 2 h for a batch of approximately 3 kg of carrots. Despite the prolonged cooking, the carrots initially remained firm, and this long cooking time was chosen to achieve a soft, nearly overcooked texture, making them easier for the horses to chew. Boiled carrots were cooled before serving.

The dried carrots (Equiherbs brand, Equiart Czesława Grycz, Kruszyna, Poland)) were purchased from an online store specializing in feed products for horses as a ready-to-eat product.

Juice was prepared from a portion of raw carrots using a SilverCrest SSJ 300 C1 300 W slow juicer (Lidl Stiftung & Co. KG, Bochum, Germany) at 60 rpm. The obtained juice was clear, with an intense orange color and an aroma typical of fresh carrots.

#### 2.2.2. Carrot Feeding

The tests were conducted in the evening, between 5 p.m. and 8 p.m. Horses participated before their evening feeding of concentrate feed, but they always had ad libitum access to roughage (hay). No feed restriction was applied prior to testing.

Different forms of carrots were served in five identical blue dishes, each with a volume of 6 L and a diameter of 32 cm. The bowls were thoroughly rinsed by hand under running clean water, without detergents, after each use to remove any remaining carrot residues and saliva, and to prevent any residual odor. Each preparation was accurately weighed, and each horse was served 150 g of each carrot form (raw, grated, boiled, dried, and juice), based on the fresh mass. Thus, each horse consumed 150 g of each type of carrot preparation, regardless of differences in density or water content. This study focused on assessing sensory preference and feeding behavior rather than nutrient intake; therefore, standardization to dry matter was not applied.

**Figure 1:**
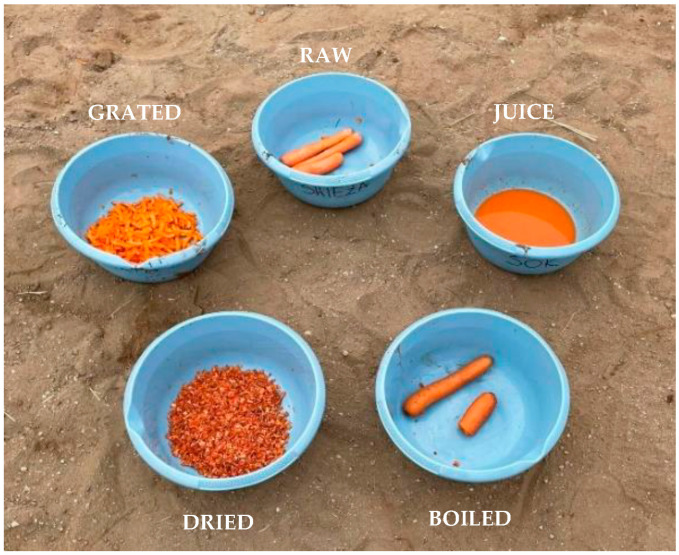
Presentation of the different forms of carrots offered to horses: raw, grated, dried, boiled and juice. Each bowl contained 150 g of the respective carrot form used during the feeding preference tests. Source: photograph by the authors.

The carrot feeding test was conducted as a cafeteria-style presentation, where all five carrot forms were offered simultaneously, allowing horses to voluntarily select between them. Dishes containing different forms of carrots were placed close together in a circular arrangement (Figure 1) to provide horses with easy access to each dish without having to walk longer distances between the dishes. The distance between the dishes was approximately 10 cm, allowing each horse to see all five carrot forms simultaneously and make a voluntary choice without being influenced by movement or repositioning. This arrangement also allowed horses to compare different forms of carrots in a single location and facilitated observations of the animals’ preferences. Dishes were placed in a close proximity to avoid displacement, which prevented horses from focusing on a single dish and enabled the researchers to record the speed at which the animals moved between different forms of carrots. During each experimental session, all horses had their bowls arranged identically to ensure standardization; however, the order of the bowls was varied between sessions to account for positional effects and potential individual side preferences, as horses may show a tendency to select feeds from their preferred side [33].

#### 2.2.3. Intake of Different Forms of Carrots

**Figure 2:**
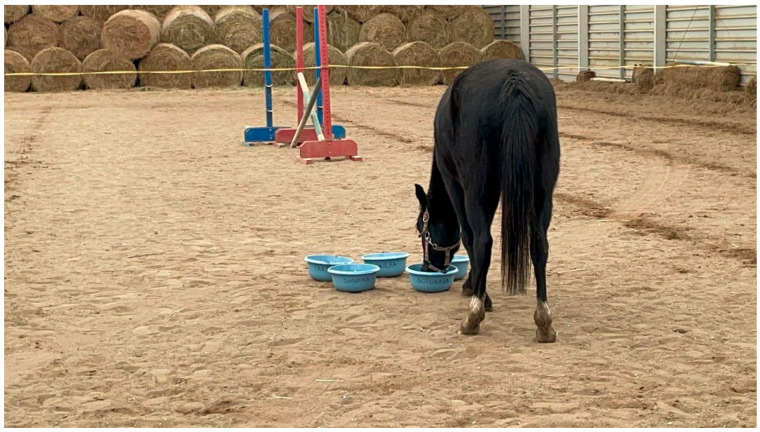
Observations of horses’ feeding behavior during the experiment. Source: photograph by the authors.

Horses’ preferences for five carrot forms (raw/unprocessed, grated, boiled, dried, and juice) were assessed individually in a designated, enclosed area of an indoor riding arena. The dishes, each containing a different carrot form, were placed in the center of the arena. Each horse was led into the arena individually and released from the lead rope immediately after entering, approximately 5 m from the bowls. Once the horse was released, the handler exited the designated area for the horse (Figure 2). All other personnel remained outside the horse’s area, approximately 10 m from the bowls, consistently maintaining silence and minimal movement, with no interaction or attempts to draw the horse’s attention to avoid distraction or influencing its behavior. Observation sessions lasted 5–10 min, ending when all dishes were emptied or the horse showed no interest for at least 30 s.

Due to logistical and financial constraints, some horses participated in three sessions, while others participated in four sessions, conducted weekly in the evening (between 5 p.m. and 8 p.m.). No other animals were present in the horse’s area, except for a mare participating in this study together with her unweaned foal, which was held on a lead rope outside the designated area for the horse consuming the carrots and did not participate in the experiment.

All sessions were video-recorded in Full HD using an iPhone 12 (Apple, Zhengzhou, China), with the camera positioned to capture both the horse and all dishes. Video recordings were analyzed by a single observer using Windows Media Player (Microsoft Corporation, Redmond, WA, USA) to ensure consistent interpretation and avoid inter-observer variation. From the video footage, we determined the order in which each carrot form was selected (e.g., raw first, grated second, boiled third) and measured the time (in seconds) spent consuming each carrot form. In cases where a horse did not consume or partially rejected a carrot form, that repetition was excluded. These measurements provided objective indicators of feeding behavior, allowing for analysis of first-choice selection and relative preference between carrot forms.

#### 2.2.4. Statistical Analysis

The results were processed statistically using the Statistica 13.3 program (TIBCO Software Inc., Palo Alto, CA, USA, 2017), except for selected tests that were performed in R software (R version 4.5.1; R Core Team, 2025, r-project.org, Vienna, Austria).). Before statistical analysis, the distribution of continuous variables was assessed for normality using the Shapiro–Wilk test. The data did not follow normal distribution; therefore, non-parametric tests were applied.

The horses’ preferences for different forms of carrots were assessed with the chi-square test and Fisher’s exact test since these methods do not assume normal distribution and are recommended for studies with small sample sizes [38]. Chi-square and Fisher’s exact test statistics with the corresponding *p*-values were calculated using R software for statistical analysis.

The feeding time for each carrot form and the order in which different forms of carrots were consumed were compared using the Friedman test for repeated measures. When significant differences were identified, Dunn’s post hoc test with Bonferroni correction was applied. The relationships between horse age vs. feeding time and the order in which different forms of carrots were selected were assessed by Spearman’s rank correlation analysis. The attractiveness of carrot juice was evaluated based on dichotomous data (tried vs. did not try carrot juice) using Cochran’s Q test, and paired data were compared by McNemar’s test.

## 3. Results

### 3.1. Time Spent Feeding on Different Forms of Carrots

The Friedman test revealed that the form of carrots offered to the horses significantly affected the time spent consuming each carrot form (*p* < 0.001). The amount of time spent feeding from bucket was shortest for boiled carrots and longest for dried carrots. Dunn’s post hoc test (with Bonferroni correction) showed that boiled carrots were eaten for a significantly shorter time than the other forms, while dried carrots were consumed for a significantly longer time relative to the other forms. No significant differences were observed between raw and grated carrots. The detailed results are presented in Table 1.

Spearman’s rank correlation analysis revealed no significant relationships between horse age and time spent consuming different carrot forms.

### 3.2. Differences in Selection Order Among Carrot Forms

The order in which different forms of carrots were selected by horses was analyzed using the Friedman test for repeated measures, which revealed that the form in which carrots were presented significantly influenced their appeal to horses (*p* < 0.01). Dunn’s post hoc test with Bonferroni correction demonstrated that raw, grated, and dried carrots were selected significantly earlier than boiled carrots, whereas no significant differences were observed among raw, grated, and dried carrots. These findings indicate that boiled carrots were the least attractive to horses.

Chi-square and Fisher’s exact tests, conducted as complementary analyses, revealed significant deviations from a random order of selection (*p* < 0.05 in all trials, *p* < 0.01 in three trials), confirming that the horses’ choice order reflected specific preferences rather than random selection.

The mean order of selection for each carrot form, along with significant differences identified by Dunn’s post hoc test, is presented in Table 2.

Spearman’s rank correlation analysis revealed no significant relationships between horse age and the order in which the analyzed forms of carrots were selected (*p* > 0.05).

### 3.3. Attractiveness of Carrot Juice

The results of Cochran’s Q test revealed that the appeal of carrot juice differed across observation trials (*p* < 0.01). None of the examined horses drank juice in the first and second trials. Carrot juice was consumed by one horse (≈5%) in the third trial and by five horses (≈24%) in the fourth trial. These observations suggest that carrot juice attracted growing interest in successive trials, although its appeal remained low, as only five out of 21 horses (≈24%) tried carrot juice during the entire experiment. Carrot juice was consumed infrequently; therefore, it was regarded as non-representative and was not included in the statistical analyses of consumption time and selection order.

## 4. Discussion

Carrot is a popular component of equine diets. Although readily digestible, raw carrots may present a feeding challenge for older horses [6] and are also difficult to store because they spoil easily [5]. Therefore, the form in which carrots are presented may affect their appeal to horses.

Research into horses’ dietary preferences has shown that factors such as taste, aroma, and texture play a significant role [30,39].

It should be noted that carrot preparations differed in moisture content, which may have affected apparent intake. According to previous studies, the nutrient composition of carrots varies depending on variety, cultivation method, and environmental conditions. On average, fresh carrots contain 10–14% dry matter, 0.8–1.1% crude protein, 0.1–0.3% crude fat, and approximately 9–10% total carbohydrates, of which 4.5–6.5% are simple sugars such as sucrose, glucose, and fructose. Crude fiber content usually ranges from 1.0 to 1.5%, and the average energy value is 40–45 kcal per 100 g of fresh mass [10,11,40]. Carrots are also a valuable source of β-carotene, typically providing 6–12 mg per 100 g of dry matter, together with smaller amounts of α-carotene and lutein, which contribute to their color and antioxidant properties [41,42]. Differences between organic and conventional systems, or between production regions, are moderate but measurable—organic carrots tend to have higher dry matter and carotenoid concentrations, whereas conventional ones often contain slightly more soluble sugars [43].

In the present study, all carrot forms were offered in equal fresh mass portions to reflect realistic feeding conditions, as horses are typically provided carrots on an as-fed basis. The experiment was therefore focused on sensory preference and feeding behavior rather than nutrient intake. Nonetheless, variations in dry matter and nutrient density between carrot preparations may have influenced apparent intake, and future studies could include laboratory dry matter standardization to quantify these effects more precisely.

The relatively low acceptance of the boiled carrot and juice forms may be explained by processing-related changes that alter the sensory characteristics of the feed. Thermal treatment during boiling can cause the loss of volatile aroma compounds and soften the carrot texture, thereby reducing oral stimulation and overall palatability. Likewise, juicing removes the fibrous structure and decreases tactile feedback during chewing, while diluting the naturally sweet flavor due to the loss of solid components. These processes likely diminish both gustatory and olfactory cues that horses use to evaluate feed. As a result, the boiled and juice forms offered less sensory complexity and lower feeding motivation compared to raw or grated carrots. This observation aligns with previous studies showing that processing and moisture alterations can significantly modify the flavor and acceptance of feedstuffs in horses [30,44].

The present findings on voluntary intake and feeding rate can also be interpreted in the broader context of equine time–activity budgets. In the present study, dried carrots were consumed more slowly than the other forms, likely because their dry texture required increased chewing and saliva production to form a bolus before swallowing, rather than reflecting lower overall palatability. Although the portion size was relatively small and not intended to measure absolute intake, this study allowed for a preliminary assessment of how different carrot forms may influence the pace of chewing. This is relevant for designing feeding practices that support horses’ natural behavioral needs, such as slow, prolonged consumption of feed. Recent evidence synthesized by Lamanna et al. [45] demonstrated that the proportion of daily time devoted to feeding varies markedly between management systems, with free-ranging horses spending approximately 56% of their time feeding, compared with only about 38% in stabled horses. Similar differences were observed between grouped and isolated animals (54% vs. 39%), and between grazing and hay-fed horses (56% vs. 39%). These results highlight that feeding behavior is sensitive to both environmental and management conditions. In this light, the differences in feeding time observed in our study depending on the carrot form may have practical implications: providing feed in a physical form that encourages longer chewing and natural foraging behavior could help extend feeding duration in stabled horses, thereby supporting their behavioral needs and welfare. Thus, our results complement the findings of Lamanna et al. [45] by demonstrating that not only the availability of feed but also its physical characteristics can influence feeding dynamics and potentially contribute to improving management practices in domestic horses.

The storage method significantly affects the shelf life and nutritional value of carrots [46]. A wide array of nutrients, including heat-sensitive vitamins, is effectively preserved in raw carrots [47]. However, raw carrots have a limited shelf life and should be stored in a cool, dry, and well-ventilated place when intended for animal feeding. In larger-scale feeding systems, carrots are often kept in shaded storage rooms or root cellars at temperatures close to 0–4 °C to minimize spoilage and maintain palatability. In stable conditions, however, long-term storage of carrots may be problematic due to variable climatic conditions throughout the year and the frequent presence of rodents in stables and storage facilities. Unprocessed carrots are susceptible to dehydration, wilting, and microbial growth, which limits their suitability for prolonged storage [48].

Boiling can temporarily improve the microbiological safety of carrots by inactivating enzymes and reducing surface microflora [49]. This treatment can increase the bioavailability of β-carotene in carrots by disrupting the structure of plant cells [50]. However, thermal processing also causes considerable losses of water-soluble vitamins, mainly vitamin C, and alters the texture and flavor of vegetables [51]. Boiled carrots are not suitable for long-term storage, as they have high moisture content and are prone to rapid microbial spoilage; therefore, they should be consumed shortly after preparation. Despite these limitations, boiled carrots may be useful for feeding horses with dental defects, as their softened texture facilitates chewing and swallowing.

Drying extends the shelf life of carrots by reducing water activity and inhibiting microbial growth, making dried carrots a stable option for feed [52,53,54]. Dried root vegetables, such as carrots, present both advantages and limitations when used as feed for horses. Their extended shelf life and reduced water activity decrease the risk of microbial spoilage, making them a practical option for long-term storage and large-scale feeding [52,55]. Additionally, drying preserves most nutrients, including fiber and carotenoids, although some heat-sensitive vitamins may be partially lost [52,56,57]. On the other hand, the increased hardness of dried roots may pose a challenge for older horses or those with dental problems, potentially limiting intake [26,58]. The altered texture and flavor can also reduce immediate palatability, requiring gradual introduction [30,59]. Similar considerations apply to other dried root vegetables, such as beetroot, where preservation extends shelf life but can modify sensory characteristics, affecting preference [56,57].

Carrot juice, particularly non-pasteurized, has a short shelf life and contains minimal dietary fiber due to the removal of insoluble fractions during juicing [47]. In this study, freshly squeezed juice was offered few hours after preparation, which may explain the initially low acceptance by horses.

It should be noted that the experimental design incorporated repeated weekly sessions, with each horse participating in only three or four observation trials depending on logistical availability. Although a separate habituation phase was not implemented, the repeated-exposure structure allowed for gradual adaptation across sessions. Nevertheless, individual variation in neophobia and previous feeding experiences may still have influenced the initial feeding responses and must be considered when interpreting the results.

This was particularly evident in the case of carrot juice. None of the horses consumed the juice during the first two sessions; consumption began only in the third trial for one horse and in the fourth trial for five horses. Initially, the juice was sniffed but not ingested, likely due to its atypical texture, as horses are known to be reluctant to consume feeds lacking appropriate tactile characteristics [39]. The gradual increase in acceptance across sessions suggests that repeated exposure facilitated familiarization. The low acceptance of juice may also reflect individual behavioral differences, as temperament and lateralization can strongly influence feed palatability and selection behavior [33]. Thus, some horses may have avoided novel or highly processed forms primarily due to sensory unfamiliarity or neophobia rather than intrinsic properties of the feed.

Therefore, a key limitation of this study is the horses’ limited familiarity with the experimental carrot forms, as they had only been exposed to raw, unprocessed carrots prior to this study. The initially low acceptance of several processed forms—especially juice—may in consequence reflect neophobia rather than true preference. Although avoidance decreased with repeated exposure, consistent with the work of Van den Berg et al. [31] and Vinassa et al. [33], the small number of sessions restricted the degree of habituation that could occur. Further limitations include the small sample size, lack of distinct age and breed groups, and a limited number of replications. Individual behavioral differences and prior feeding experiences likely influenced feeding responses and could not be fully controlled under the current design. Finally, chewing efficiency and dental condition were not assessed, despite their potential importance for feed selection and intake, particularly in older horses.

In summary, the present study confirmed that the form in which carrots are offered to horses affects not only their acceptability, but also feeding efficiency and animal safety. Feedstuffs differ in texture, shelf life, and nutritional value and they should be selected according to the age and health status of animals to support their welfare.

Future research could address these gaps by examining the interactions between age, dental health, and feed texture in a larger and more diverse cohort of horses. Systematic evaluation of dental status, monitoring of chewing behavior, and quantitative measures such as intake rate, time spent feeding, and preference ranking could be combined with behavioral observations to provide a more comprehensive assessment of how feed form affects feeding efficiency and welfare. Standardizing dry matter and nutrient composition across different carrot preparations would also clarify the contribution of sensory and nutritional factors to feed selection. Such studies would strengthen methodological rigor and offer practical guidance for equine practitioners and owners in selecting carrot forms that best support intake and welfare.

## 5. Conclusions

The results of this study indicate that the form of carrots offered to horses influences feeding behavior. A key finding of our study is that feed intake time differed between carrot forms, with boiled carrots consumed most quickly and dried carrots most slowly. Horses were initially reluctant to consume carrot juice; while acceptance increased over successive trials, it remained generally low, indicating limited appeal despite some habituation. Raw carrots were generally selected first, reflecting high sensory appeal, while boiled carrots were the least preferred solid form. No significant effect of horse age on feeding time or selection order was observed. It should be noted, however, that the horses’ limited familiarity with the experimental carrot forms may have influenced their choices, representing a limitation of this study. Overall, these findings suggest that carrot form can affect voluntary intake and feeding dynamics, which has implications for diet planning, feeding efficiency, and equine welfare, particularly in stabled or older horses with dental challenges.

## Figures and Tables

**Table 1:** Time spent feeding (in seconds) on different forms of carrots.

	Form of Carrots			
	Raw	Grated	Boiled	Dried
*N*	69	71	59	65
$\overset{¯}{x}$	79.1 ^a^	80.4 ^a^	43.2 ^b^	94.3 ^c^
SD	±31.2	±35.0	±25.4	±50.7
Min	12.0	16.0	5.0	12.0
Median	78.0	76.0	38.0	86.0
Max	157.0	177.0	140.0	254.0

The number of observations (*N*) represents the valid cases included in the analysis for each carrot form. Observations in which a horse did not consume a particular form were excluded. $\overset{¯}{x}$ = mean; SD = standard deviation; Min = minimum; Median = median; Max = maximum. Mean values marked with the letters ^a^, ^b^, and ^c^ differ significantly (*p* < 0.05).

**Table 2:** Mean order of selection/interest in different forms of carrots based on the results of the Friedman test and Dunn’s post hoc test.

Form of Carrots	Mean Order (1–5) ± SD
Raw	2.2 ^a^ ± 0.9
Grated	2.3 ^a^ ± 0.9
Boiled	2.9 ^b^ ± 0.8
Dried	2.4 ^a^ ± 0.9

Mean values marked with the letters ^a^ and ^b^ differ significantly (*p* < 0.05).

## Data Availability

The data that support the findings of this study are available from the corresponding author upon reasonable request. Video recordings are retained by the authors and are not publicly available online.
